# Short hairpin RNA- mediated gene knockdown of FOXM1 inhibits the proliferation and metastasis of human colon cancer cells through reversal of epithelial-to-mesenchymal transformation

**DOI:** 10.1186/s13046-015-0158-1

**Published:** 2015-05-03

**Authors:** KanKan Yang, LinHua Jiang, You Hu, Jing Yu, HenFeng Chen, YiZhou Yao, XinGuo Zhu

**Affiliations:** Department of General Surgery, The First Affiliated Hospital of Soochow University, 215006 Suzhou, Jiangsu Province China; Department of Laparoscopic Surgery, The First Affiliated Hospital of Soochow University, 215006 Suzhou, Jiangsu Province China

**Keywords:** FOXM1, EMT, Metastasis, Colorectal cancer

## Abstract

**Background:**

The Forkhead box M1 (FOXM1) is an oncogenic transcription factor and plays a significant role in cell EMT, proliferation, metastasis in a multitude of human solid tumors including colorectal cancer (CRC). However, the underlying molecular mechanisms by which FoxM1 contributes to epithelial-to-mesenchymal (EMT) and metastasis have not been fully elucidated in CRC.

**Methods:**

In our study, we investigated FOXM1 protein expression in 87 CRC tissue specimens, invasive lymph nodes and adjacent paired normal colorectal tissues by immunohistochemical analysis. Then we transfected FOXM1 specific shRNA into SW620 cells to examine effect of FOXM1 on proliferation, colony formation, migration and invasion in vitro. Western blotting and real-time PCR were used to detect the protein and mRNA expression of FOXM1 and EMT-related markers.

**Results:**

FOXM1 was overexpressed in CRC tissues, invasive lymph nodes and CRC cell lines. FoxM1 overexpression was significantly associated with lymph node metastasis (P < 0.001), and tumor recurrence (P < 0.001). Moreover, downregulation of FOXM1 in SW620 cells by shRNA approach inhibited cell growth, clonogenicity, migration and invasion in vitro. In addition, decreased FOXM1 expression in SW620 cells reversed the acquisition of EMT phenotype by up-regulating E-cadherin, as well as reduction Vimentin and Snail expressions at protein and mRNA levels.

**Conclusions:**

FOXM1 may regulate CRC cells metastasis through EMT program and FOXM1 may be a potential target for treatment of CRC.

## Background

Colorectal cancer (CRC) is the third most common cancer and the third leading cause of cancer death in men and women in the United States. Although early detection tests and treatments have been improved in clinical practice, including modified surgical techniques and neoadjuvant chemotherapy combined with radiation therapy in CRC patients, the 5-year survival rate is decreasing to 12.5% in the advanced CRC patients who have metastasis of distant organs [1-3]. Therefore, there is an urgent need to identify novel prognostic hallmarks and to improve on current understanding of the molecular mechanisms of advanced CRC.

The transcription factor Forkhead box M1 (FOXM1) is an oncogenic transcription factor belongs to the FOX protein super family that shares an evolutionarily conserved winged helix DNA-blinding domain [4,5]. Large-scale gene expression analysis by means of microarrays have demonstrated that FoxM1 is one of the most common overexpressed genes in a multitude of human solid tumors [6], including hepatocellular carcinomas [7], pancreatic cancer [8], breast cancer [9], ovarian cancer [10], colorectal cancer [11] and lung cancer [12], suggesting that FOXM1 is essential to regulate the tumorigenicity. Many studies have reported that FOXM1 is known as a key regulator of the cell cycle by regulating the transition from G1 to S and G2 to M phase and mitosis [13,14], playing a positive effect on cell proliferation. Futhermore, enhanced expression of FoxM1 is associated with advanced stage, lymph node matastasis and acts as an independent prognostic factor in non-small cell lung cancer (NSCLC) [15]. Beyond that cell proliferation, FOXM1 also plays important roles in tumor angiogenesis, EMT, invasion, and metastasis [9,16-20]. The actual occurrence of EMT serves as a dominant role in invasion and metastasis of colon cancer [21], which is regulated by a various signal pathways, such as FOXM1-PLAUR [22], FOXM1-caveolin-1 signaling pathway [23]. Emerging evidences suggest that enhanced FoxM1 levels lead to the acquisition of EMT phenotype, which contributes to tumor cell aggressiveness along with a series of molecule changes of epithelial or mesenchymal markers [24]. On the contrary, for example, downexpression of FOXM1 in RNAi-mediated gastric cancer cells reversed the EMT phenotype and upregulated the expression of epithelial markers E-cadherin, as well as downregulated the expression of mesenchymal markers ZEB1, ZEB2 and Vimentin [25]. However, the precise function and internal mechanisms of FOXM1 in colorectal cancer cells EMT and metastasis remain still indistinct.

In our present study, we detected the expression of FOXM1 in colorectal tumor tissue specimens by immunohistochemical staining from 87 CRC patients and investigated the relationships among mediated gene knockdown of FOXM1 on SW620 cells and EMT, proliferation, migration and invasion in vitro. Our results show that the downregulation of FoxM1 inhibits the cell migration, invasion, and proliferation of SW620 cells and reverses the EMT phenotype by up-regulating epithelial cell markers E-cadherin, as well as down-regulating the expression of the mesenchymal cell markers Vimentin and Snail at protein and mRNA levels. The results provide supportive evidence that FOXM1 may be an effective therapeutic target in CRC.

## Materials and methods

### Human colorectal cancer tissues and colon cancer cell lines

Human colorectal cancer tissues were obtained from 87 patients at the Department of General Surgery, the First Affiliated Hospital of Soochow University from 2008-2013. Each tumor tissue and adjacent normal colon tissue (at least 2cm distance from the tumor site) were collected from the same patient with a clear histological diagnosis of CRC who had received no any therapy before sample collection. The researches were supported by the Independent Ethics Committee (IEC) of the First Affiliated Hospital of Soochow University and all patients were provided written informed consent. Human colon cancer cell lines HCT116, SW620, SW480, LOVO and DLD-1 were purchased from the Chinese Academy of Sciences (Shanghai, China). All five cell lines were maintained in DMED supplemented with 10% fetal bovine serum (Sijiqing Biological Engineering Materials Co., Hangzhou, China) and cultured at 37°C in a humidified atmosphere containing 5% CO_2_.

### Immunohistochemistry (IHC)

The protein of FOXM1 of 87 human colorectal cancer tissue samples and adjacent normal colorectal tissues was detected through immunohistochemical staining (IHC). IHC was performed using the streptavidin-peroxidase coujugate method. Surgical specimens were fixed in 10% formalin and embedded in paraffin. Briefly, the paraffin-embedded tissues were serially cut into 4μm sections, dewaxed, and rehydrated. Sections of paraffin-embedded tissues were then blocked with peroxide in methanol, and nonspecific immunoglobulin binding was blocked by incubation with 10% normal goat serum for 15min. After rinsing with PBS, the sections were incubated at room temperature for 1h with Foxm1 polyclonal rabbit-anti-human antibody (Abcam, UK) at 1:200 dilution. After a PBS rinse, slides were then incubated for 25min at room temperature with biotinylated goat-anti-rabbit immunoglobulin (1:1000, Zhongshan Biotechnology, China) followed by incubation with peroxidase-conjugated streptavidin for 20min. Finally, the slides were stained with fresh 0.05% 3, 3′-diaminobenzidine (DAB), counterstained with hematoxylin, dehydrated, cleared in xylene, and fixed. Histological assessment was performed as described previously [26]. Immunostaining was independently examined by two clinical pathologists who were blinded of the patient outcome. Five high-power fields (400 × magnification) were randomly counted for each section. The brown staining on the cytoplasm was read as positive reactivity for FOXM1. The presence of brown colored granules on the cytoplasm was taken as a positive signal, and was divided by color intensity into not colored, light yellow, brown, tan and is recorded as 0, 1, 2, 3, respectively. We also chose five high-power fields from each slice and scored them. Positive cell rate of < 25% was a score of 1, positive cell rate of 25 ~ 50% was a score of 2, positive cell rate of 51 ~ 75% was a score of 3, positive cell rate of >75% was a score of 4. The scores for FOXM1 positivity and staining intensity were multiplied to obtain a final score, which determines FOXM1 expression as (− = 0; + =1-4; ++ = 5–8; +++ = 9–12). In our current study, we classified all of the samples into the high expression group (++ or +++) and the low expression group (− or +) according to the protein expression.

### Short hairpin RNA transfection of human colon cancer cell line SW620

Human FOXM1 shRNA (5′-GGACCACUUUCCCUACUUU-3′) and control-shRNA (5′-GGACCUGUAUGCGUACAUU-3′) were synthesized by GenePharma (shanghai, china). SW620 cells were transfected with shFOXM1 or control-shRNA using Lipofectamine 2000 (Invitrogen, Life Technologies), according to the manufacturer’s instructions.

### Quantitative real-time reverse transcription PCR (QRT-PCR)

The mRNA expression of FOXM1, E-cadherin, Vimentin and Snail in SW620 cells after FOXM1-shRNA transfection were quantified by real-time RT-PCR. Total RNA was extracted from cells and tumor tissues using TRIzol Reagent (Invitrogen, Life Technologies) and cDNA was synthesized from 2μg of RNA using the First Strand cDNA Synthesis Kit (Fermentas) according to the manufacturer’s instructions. QRT-PCR was carried out using Power SYBR® Green PCR Master Mix (ABI, USA) on the 7500 real time PCR system (ABI, life technology). The β-actin was used as a loading control for each specific gene. Each experiment was performed three times and each sample was tested in triplicate. The sequences for sense (S) and antisense (AS) primers as follows: human-foxm1-S, 5′-GGAGGAAATGCCACACTTAGCG-3′, human-foxm1-AS,5′-TAGGACTTCTTGGGTCTTGGGGTG-3′, human-E-cadherin-S, 5′-CGGGAATGCAGTTGAGGATC-3′, human-E-cadherin-AS,5′-AGGATGGTGTAAGCGATGGC-3′, human-Vimentin-S,5′-GAGAACTTTGCCGTTGAAGC-3′, human-Vimentin-AS,5′-GCTTCCTGTAGGTGGCAATC-3′, human-Snail-S,5′-CTCTTTCCTCGTCAGGAAGC-3′, human-Snail-AS,5′-GGCTGCTGGAAGGTAAACTC-3′, β-actin-S,5′-CCACACTGTGCCCATCTACG-3′, β-actin-AS,5′-AGGATCTTCATGAGGTAGTCAGTCAG-3′. The PCR conditions consisted of 5min at 95°C 1cycle, 30sec at 95°C, 30sec at 55°C, 30sec at 72°C and 7min at 72°C 40cycles. The 2^-ΔΔC^_T_ method was applied to analyze the relative changes in gene expression [27].

### Protein extraction and western blot analysis

Whole protein extracts from SW620 at 72h following shRNA transfection or untransfection were lysed in ice-cold RIPA lysis buffer (Beyotime Inc., NanTong, China) according to manufacturer’s protocol. From each sample preparation 50μg of whole protein was separated by SDS-PAGE and then transferred to PVDF membranes (Millipore, USA). Standard Western blotting was performed using a polyclonal rabbit antibody against human FoxM1 (1:1000, Abcam, UK), mouse anti-human E-cadherin (1:1000, Abcam, UK), rabbit anti-human Vimentin (1:1000, Abcam, UK), rabbit anti-human Snail (1:500, Abcam, UK) and rabbit anti-β-actin (1:1000., Beyotime, china). The signals from the primary antibody was amplified by HRP conjugated anti-mouse IgG or anti-rabbit IgG (1:1000; Beyotime, china) and detected with Enhanced Chemiluminescence Plus kit (Beyotime, China).

### MTT assays of cell viability

Briefly, 24hours after transfection, SW620 cells of three groups were digested, re-suspended and seeded at a density of 4 × 10^3^ cells/well in 96-well culture plates, After 24h,48h and 72h of incubation in complete medium, cells were added 20μl MTT solution (5mg/ml) at 37°C for 4hours. Then supernatants were removed and formazan crystals were dissolved in 150μl DMSO. After gentle shaking for 10 minutes, the absorbance (A) at 490nm was measured by using a microplate reader. Each sample was four replicate wells and the experiment was repeated three times.

### Clonogenic assays

Clonogenic assay was conducted to examine the effect of FoxM1-shRNA on cell growth in SW620 cells, as described previously [9]. 4 × 10^5^ SW620 cells were plated in a 6-well plate. After 24h of transfection, the cells were trypsinized, and 1,000 single viable cells were plated in three 6-well plates. The cells were then incubated for 14days at 37°C in the condition of 5% CO_2_/5% O_2_/90% N_2_. Colonies were stained with 0.1% crystal violet, washed with water, and counted ten random fields manually. The colonies containing at least 100 cells were scored. The surviving fraction in FoxM1-shRNA transfected SW620 cells was normalized to untreated control cells with respect to clonogenic efficiency.

### Wound healing assay

Wound healing assay was adopted to test the migration ability of colon cancer cells. In our study, SW620 cells were digested after transfection by specific shRNA and control shRNA to human Foxm1 for 24h in 6-well plates, 2 × 10^5^ cells were plated in 24-well plates, when cell confluence reached approximately 100%, the old medium was removed and the monolayer was wounded by scratching with a 10-μl sterile pipette tip lengthwise along the chamber, then cells were washed three times with PBS and cultured with serum-free medium at 37°C. Images of cells migrating into the wound were photographed at 0h, 24h, 48h and 72h using an inverted microscope. Wound width (μm) was measured using OpenLab software. Wound healing rate = (0h scratch width - 24h/48h or 72h scratch width)/0h scratch width × 100%. The experiments were repeated three times.

### Cell migration and invasion assays

The migratory and invasive activity of the FoxM1 or control shRNA-transfected SW620 cells was tested using the Transwell chambers equipped with a pore size of 8μm (Corning, USA) according to the manufacturer’s recommendations. After 24h of transfection, 2 × 10^4^ SW620 cells per well were resuspended in serum-free DMEM medium and seeded into the Transwell inserts either uncoated (for migration assay) or coated (for invasion assay) with growth factor-reduced Matrigel (BD Biosciences, Bedford, MA), whereas the lower chambers were filled with 500μl DMEM with 10% FBS. After 24h incubation at 37°C, the cells on the upper side of the insert filter were completely removed by wiping with a cotton swab, and the cells that had invaded were fixed in methanol and stained with 0.1% crystal violet. The cells were counted manually under an inverted microscope on five random fields (scale bar =200μm). Each experiment was repeated in triplicate.

### Statistical analysis

Data are expressed as the means ± standard deviation (SD). Significant differences between the groups were determined using the student’s *t*-test and Chi-square test. A value of P < 0.05 was considered to indicate a statistically significant difference. All statistical analyses were performed with SPSS17.0 software (SPSS Inc, Chicago, IL, USA).

## Results

### FoxM1 is over-expressed in human colorectal cancer patients

**Figure 1:**
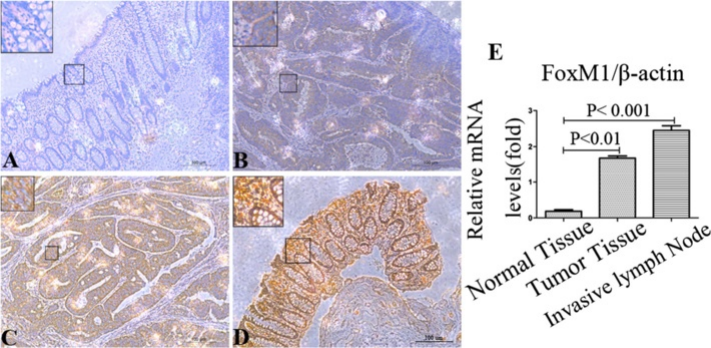
FoxM1 is highly expressed in human CRC patients. **(A-D)**: Foxm1 protein expression in CRC cells, normal colon cells and invasive lymph nodes from immunohistochemical staining. The protein expression of FOXM1 was negative **(A)**, mild positive **(B)**, positive **(C)**, strong positive **(D)**, respectively. Scale bar = 100μm. **(E)**: QRT-PCR analyses of colon cancer tissues, invasive lymph nodes and adjacent normal tissues from 4 CRC patients. Foxm1 mRNA expression levels were significantly higher in colon cancer tissues and invasive lymph nodes than paired adjacent normal colon tissues.

To investigate whether FoxM1 is highly expressed in CRC tissues, we first detected the expression of FoxM1 protein in the 87 primary colorectal tumors and paired adjacent normal colorectal tissue specimens as well as invasive lymph nodes using immunohistochemical staining. Our immunostaining results showed a FoxM1-positive staining in the nucleus and/or cytoplasm of colorectal tumor cells and invasive lymph nodes, whereas there was negative or weakly positive staining of FoxM1 in the paired adjacent normal colorectal tissues (Figure 1A-D). To further confirm the expression of FoxM1 in colon cancer, we detected the expression of FoxM1 in fresh colon cancer tissues, metastatic lymph nodes and adjacent normal tissues from four same patients. Real-time PCR was subjected to examine the mRNA levels. Consistent with the level of FoxM1 protein expression determined using immunostaining analyses, primary colorectal tumors and metastatic lymph nodes had significantly higher levels of FoxM1 mRNA expression than did surrounding normal colorectal tissue from 4 patients by QRT-PCR analysis, the difference between them was statistically significant (Figure 1E p < 0.01). These results indicated that FoxM1 is commonly over-expressed in human colorectal tumors, particularly in metastatic lymph nodes.

### Relationship between Foxm1 expression and clinicopathologic parameters in CRC patients

To further assess the clinical relevance of FoxM1 overexpression in CRC patients, we then analyzed the relationship between the level of FoxM1 expression levels and clinicopathologic parameters in 87 CRC patients using immunohistochemical staining. As shown in Table 1, we observed that high expression of Foxm1 was significantly correlated with regional lymph nodes metastasis (*χ*^2^ = 9.184, p = 0.002) and tumor recurrence (*χ*^2^ = 8.025, p < 0.001); however, FoxM1 expression level was not associated with other clinicopathologic features, such as age, gender, tumor size, tumor location, TNM stages and histological differentiation (all p > 0.05).

**Table 1:** **Relationship between FoxM1 expression from IHC and clinicopathological factors of 87 CRC patients**

**Clinic parameters**	**Total**	**FoxM1 expression**		**χ** ^**2**^	**P value**
		**None or low**	**High**		
**Age(years)**	T=87	N=35(40.2%)	N=52(59.8%)		
<65	33(37.9%)	15(45.5%)	18(54.5%)	0.604	0.437
>=65	54(62.1%)	20(37.0%)	34(63.0%)		
**Gender**					
Male	42(48.3%)	19(45.2%)	23(54.8%)	0.847	0.357
Female	45(51.7%)	16(35.6%)	29(64.4%)		
**Tumor size**					
<5cm	56(64.4%)	20(35.7%)	36(64.3%)	1.333	0.248
>=5cm	31(35.6%)	15(48.4%)	16(51.6%)		
**Tumor location**					
Right colon	33(37.9%)	11(33.3%)	22(66.7%)	1.052	0.305
Left colon and rectum	54(62.1%)	24(44.4%)	30(55.6%)		
**TNM Stage**					
Stage I/II	42(48.3%)	19(45.2%)	23(54.8%)	0.847	0.357
Stage III/IV	45(51.7%)	16(35.6%)	29(64.4%)		
**Histological differentiation**					
Well	28(32.2%)	12(42.9%)	16(57.1%)	1.873	0.392
Moderate	46(52.9%)	20(43.5%)	26(56.5%)		
Poor	13(14.9%)	3(23.1%)	10(76.9%)		
**Lymph node metastasis**					
Yes	47(54.0%)	12(25.5%)	35(74.5%)	9.184	0.002***
No	40(56.0%)	23(57.5%)	17(42.5%)		
**Tumor recurrence**					
Yes	53(60.9%)	15(28.3%)	38(71.7%)	8.025	0.005***
No	34(39.1%)	20(58.8%)	14(41.2%)		

***P < 0.001.

### FoxM1 is up-regulated in CRC cell lines and associated directly with migration ability of colorectal cancer cells

**Figure 2:**
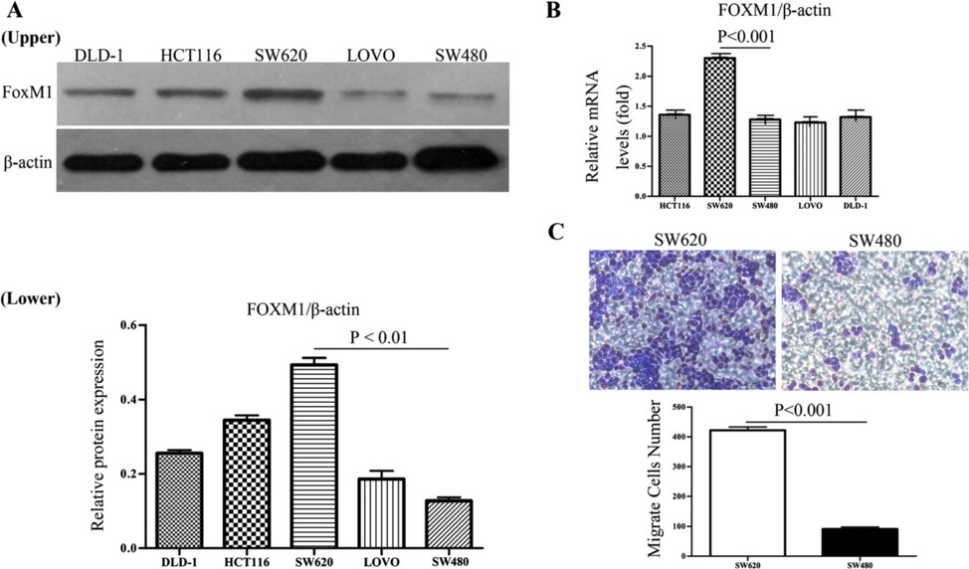
Expression of FoxM1 in CRC cell lines. **(A)**: Western blot analysis of FOXM1 protein expression in CRC cancer cell lines (Upper). The ratio between the grey levels of FOXM1 and β-actin of the each CRC cell lines through Western blotting (Lower). **(B)**: Real-time PCR analysis of FOXM1 mRNA expression in HCT116, LOVO, DLD-1, SW480, SW620 cells. **(C)**: the migration ability of SW480 and SW620 colon cancer cells. The data showed that SW620 cells presented higher levels of migration ability than SW480 cells. The experiments were repeated three times.

To evaluate the baseline expression levels of FoxM1 in a series of human colorectal cancer cell lines, including HCT116, LOVO, DLD-1, SW480, SW620, we detected the mRNA and protein expression of FoxM1 by real-time PCR and Western Blot analyses, respectively. Our studies evidenced that SW620 cells showed the highest expression of FoxM1, especially in relation to the SW480 cell line (Figure 2A&B). To further test whether the expression levels of FoxM1 were related to migration ability of CRC cells, we measured the effects of FoxM1 expression levels on cancer cell migration by transwell systems between SW480 and SW620 cells; we found that the numbers of migratory SW620 cells were more dramatically higher than those of SW480 cells (Figure 2C, P < 0.001).

### Deletion of FoxM1 gene down-regulated the mRNA and protein levels of FoxM1 expression in SW620 cells

**Figure 3:**
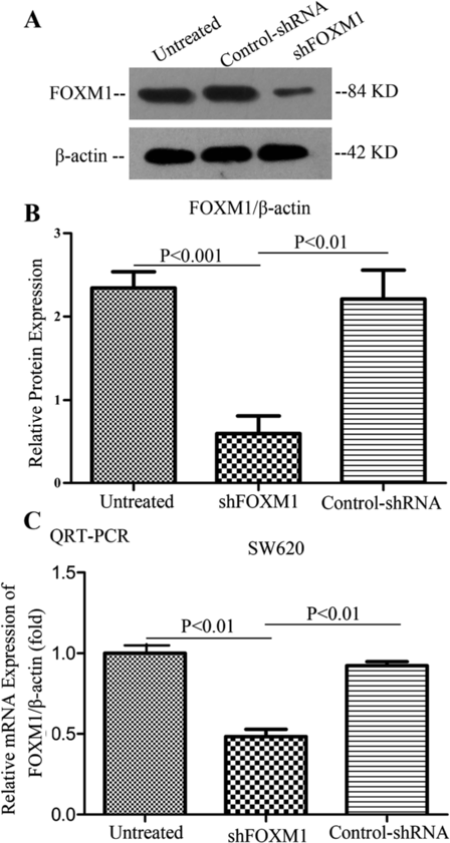
Deletion of FoxM1 gene down-regulated the expression of FoxM1 in SW620 cells by shRNA. Western blotting **(A-B)** and qRT-PCR analyses **(C)** showed that the level of FoxM1 expression was remarkably decreased in stably FoxM1-depleted SW620 cells at the mRNA and protein levels, respectively. β-actin as a loading control. Each experiment was repeated three times.

In order to explore the effect the shRNA silencing FoxM1 expression in SW620 cells, SW620 cells were transfected with the FoxM1 shRNA (shFoxM1) and control-shRNA; after 72h, the total protein and RNA of all transfected and untransfected cells were extracted and analyzed by Western blots and real-time PCR. As shown in Figure 3A-C, FoxM1 expression was distinctly decreased at mRNA and protein levels in transfected cells with FoxM1 shRNA compared with control-shRNA transfected cells and untreated cells. The results showed that the specific shRNA to FoxM1 effectively suppressed the expression of FoxM1 in SW620 cells.

### Down-expression of FoxM1 inhibited the proliferation and clonogenicity in SW620 cells

**Figure 4:**
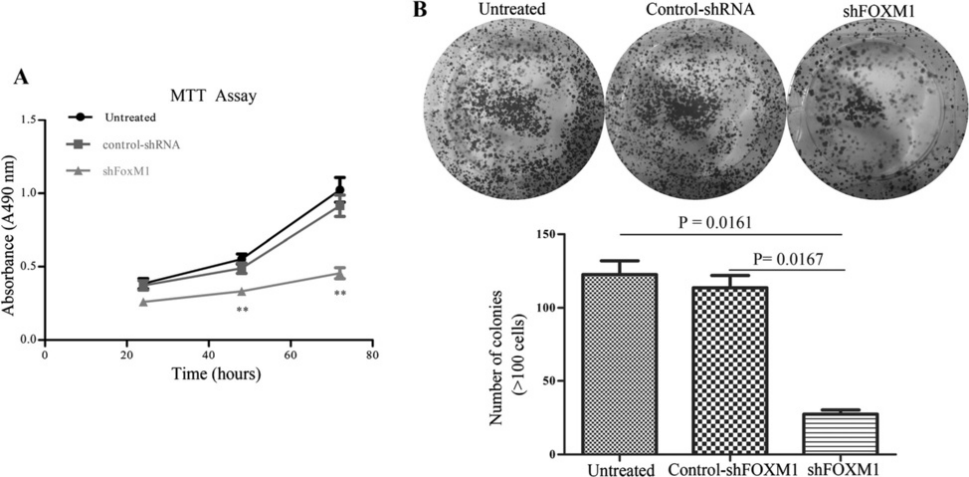
Effect of FoxM1 on the proliferation and clonogencity of SW620 cells. **(A)**: Down-regulation of FoxM1 expression inhibited the proliferation of SW620 cells compared with the Untreated cells and control-shRNA transfected cells by MTT assay. Figures are curves of SW620 cell growth after transfection for 24, 48 and 72h by MTT assays. **(B)**: Down-regulation of FoxM1 expression reduced the numbers of colonies significantly in SW620 cell line. Cell colonies were stained with 0.1% crystal violet and the colonies containing above 100 cells were counted manually. All data are presented as the means ± SD of three independent experiments.

In order to determine the effect of FoxM1 on the proliferation of SW620 cells in vitro, the proliferation curves were detected by MTT assays at 24h, 48h, 72h after 24h of transfection. We found that SW620 cells which down-regulated the FoxM1 expression experienced a significant inhibition of cell viability compared with control-shRNA and untreated cells respectively (Figure 4A, both P < 0.01).

To further examine whether the knockdown of FoxM1 gene reduces the clonogenic formation of SW620 cells, after 24h of tranfection, 2 × 10^3^/well of SW620 cells were plated in 6-well plates and cultured 14days to observe the numbers of colony formation. The results showed that the numbers of colonies in FoxM1-depleted SW620 cells displayed an apparent reduction compared with control-shRNA and untreated cells respectively (Figure 4B, p < 0.05).

### Knockdown of FoxM1 expression by shRNA induced a cellular morphologic change and EMT-related mRNA and protein expressions in SW620 cells

**Figure 5:**
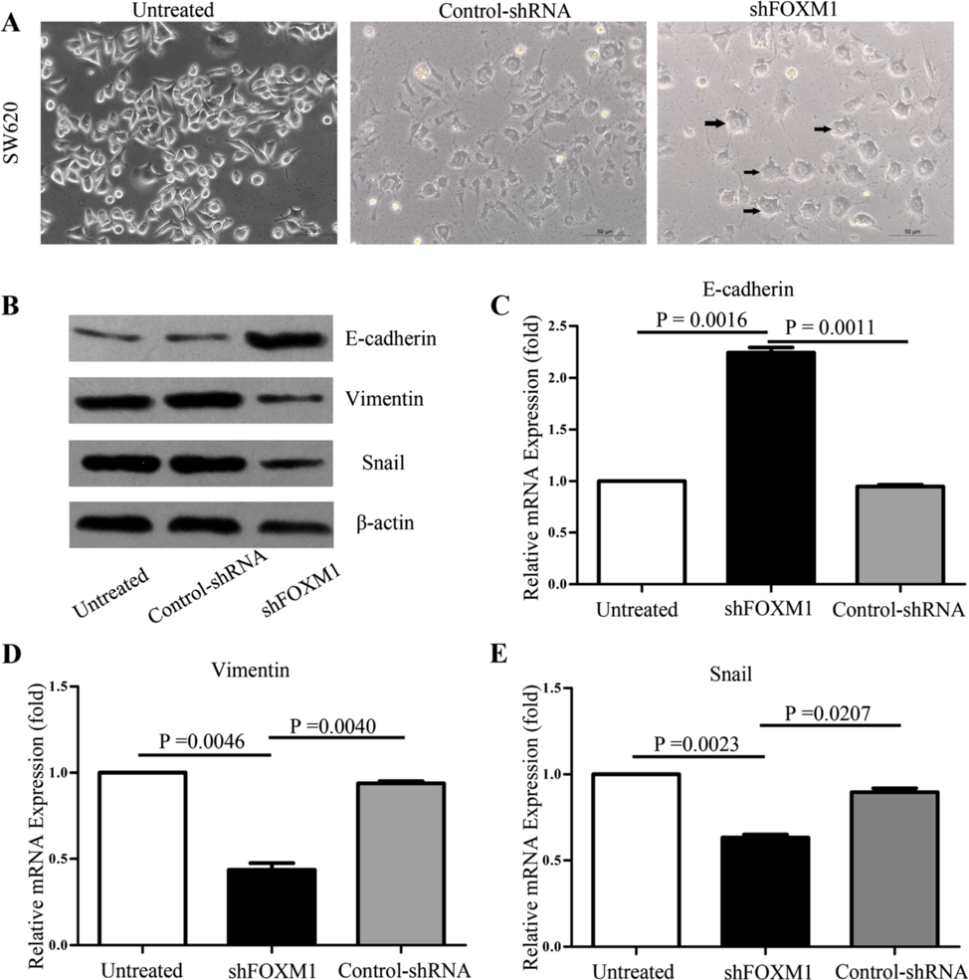
FoxM1-depleted SW620 cells underwent morphological change and effect of FoxM1 on the reversal of EMT in SW620 cells. **(A)**: SW620 cells were transfected with shFOXM1 or control shRNA and incubated for 72hours. Photomicrographs suggested SW620 cells suffered morphologic changes of mesenchymal-to-epithelial transition after knockdown of FOXM1 expression. Scale bar = 50μm. **(B)**: Expression of EMT markers was determined by western blots, β-actin as a loading control. **(C-E)**: Total RNA was extracted from transfected and untransfected SW620 cells by shFOXM1 or control shRNA and subjected to real-time PCR for E-cadherin, Vimentin, and Snail relative mRNA expression. The data were calculated with the 2^-△△C^_T_ Method.

As illustrated in Figure 5A, SW620 cancer cells have a typical mesenchymal morphology characterized by elongated, spindle-shaped phenotype. To examine whether down-regulation of FoxM1 expression could change cell morphology, we transfected FoxM1 shRNA (shFOXM1) and control shRNA into SW620 cells; after 72hours of transfection, we found that in the monolayer culture system, SW620 cellular morphology changed from an elongated, spindle-shaped, mesenchymal phenotype to a more rounded, epithelial-like phenotype after knockdown of FoxM1 expression and observation under the microscopy. The results indicated that knockdown of FoxM1 expression altered mesenchymal morphology of SW620 cells and reversed the typical epithelial-to-mesenchymal transition.

To further understand the internal molecular mechanism of mesenchymal-to-epithelial transition when FoxM1 was down-expressed, we investigated the EMT-related protein expression levels in transfected and untransected SW620 cells by western blotting (Figure 5B). The knockdown of FoxM1 gene by shRNA led to markedly reduce the mesenchymal marker Vimentin and Snail protein expression, whereas significantly increased the protein level of E-cadherin, which is a typical epithelial marker. Consistent with the real-time PCR analysis (Figure 5C-E), the down-regulation of FoxM1 expression significantly enhanced E-cadherin expression and attenuated Vimentin and Snail expression in FoxM1-knockdown SW620 cancer cells compared with those of control cells at the mRNA levels. The results demonstrated that FoxM1 played an important role in epithelial-to-mesenchymal transition regulation in CRC.

### Effect of altered FoxM1 expression on SW620 colon cancer cells migration and invasion in vitro

**Figure 6:**
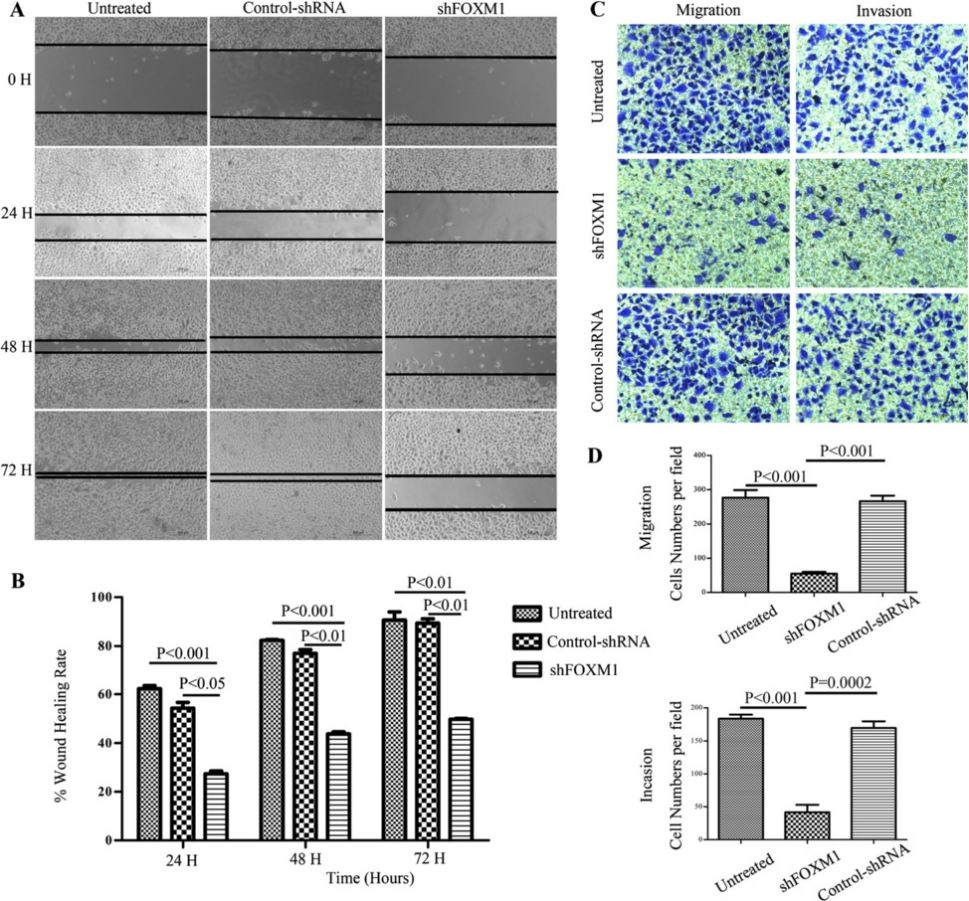
Effect of altered FOXM1 expression on colorectal cancer cell migration and invasion in vitro. **(A)**: Wound healing assays were carried out at 24h after transfection in 24-well plates, when cell confluence rate reached above 90% and a linear wound across the monolayer was done. The wound gap was photographed every 24h, the gap width was measured (μm) using Open Lab software. **(B)**: the wound rate was calculated and displayed graphically as described in the Materials and Methods. **(C-D)**: SW620 cells of three groups were digested and resuspended in serum-free culture medium and allowed to migrate toward the lower chamber with coated or uncoated matrigel for 24h. Invading cells were stained with 0.1% crystal violet and counted manually. C-Left: transwell migration assay, Right: transwell invasion assay. **(D)**: The number of invading SW620 cells by cell migration (upper) and invasion (lower) assay was counted manually. Each experiment was repeated thrice independently. Scale bar = 200μm in those figures.

Previous studies had suggested that down-expression of FoxM1 promoted E-cadherin expression and suppressed Vimentin and Snail expression, which were pronouncedly associated with EMT regulation. To determine whether attenuated FoxM1 expression reversed the EMT process and further inhibited the migration and invasion in the colorectal cancer cells, we first explored the role of FoxM1 in CRC cell migration via the wound healing assay. The FoxM1-decreasing SW620 cells showed comparatively slower migration towards the wound space; however, control shRNA cells and untreated cells migrated aggressively and nearly closed the wound at 72h after transfection (Figure 6A-B, ** p < 0.01). Similar results arosed in the transwell migration system (Figure 6C-left, D-upper, *** P < 0.001).

Consistent with the impact of altered FOXM1 expression on the invasion of SW620 cancer cells in vitro, transwell invasion assay revealed that FoxM1 shRNA-transfected cells showed a low level of penetration through the Matrigel-coated membrane compared with the control cells (Figure 6C-right, D-lower, *** P < 0.001). All these results clearly show that FoxM1 participates in EMT process and promotes the migration and matastasis in colorectal cancer cells.

## Discussion

FOXM1 is commonly regarded as an oncogenic transcription factor and abnormal expression and activation of FOXM1 is associated with the proliferation and metastasis of human colon cancer cells, as an independent poor prognostic factor and conversely correlated with poor OS and MFS in CRC patients [11,17,28]. In the present study, FOXM1 protein expression was examined using immunohistochemisty analysis in CRC patients and we detected that FOXM1 was highly expressed in most human primary CRC tissues, particularly in invaded lymph nodes, whereas lowly expressed in adjacent normal colon tissues. Meanwhile, the strong relationships among FOXM1 expression and clinicopathology features had been investigated in CRC patients by IHC, our results indicated that FOXM1 overexpression was significantly associated with regional lymph nodes metastasis and tumor recurrence, suggesting important roles of FOXM1 in human colorectal cancer tumorigenesis and distant metastasis. The results show that FOXM1 has the potential to be a novel therapeutic target in CRC.

A mounting body of evidence notes that FOXM1 is a critical regulator of both the G1/S and G2/M transitions through the cell cycle progression, which is required for proliferative expansion during tumor progression [5]. Knockdown of FoxM1 inhibits expression of JNK1 and cyclinA2, which are involved in G1-S progression and in accumulation of cells in G2/M [29]. Previous research had revealed the role of FOXM1 in cell growth; increased expression of Foxm1 in Rosa26-Foxm1b transgenic mice directly regulated the cell-cycle progression of colon tumor cells by promoting S-phase progression and entry into mitosis [30]. Consistently, our current studies suggested that inhibition of FOXM1 expression in SW620 cells by shRNA transfection decreased the proliferation and colony forming capability compared with that control-shRNA SW620 cells. Besides the cell proliferation capability of FOXM1, aberrant regulation of FOXM1 is a leading factor of malignancy tumor metastasis [10]. In our five CRC cell lines, HCT116, SW620, SW480, LOVO, DLD-1, western blot and qRT-PCR analyses showed that FOXM1 expression levels were significantly increased, especially in SW620 cells. Meanwhile, we found that SW620 cells exhibited the much higher metastatic ability, revealing that FOXM1 overexpression may promote the tumor metastasis.

As shown in our wound healing assays and transwell migration/invasion systems, down-expression of FOXM1 remarkly inhibited the wound healing, migration and invasion of FOXM1-depleted SW620 cells. All our in vitro assays suggest a pivotal role for the FOXM1 in the metastasis progression of colorectal cancer and FOXM1 silencing could efficiently control the CRC cells metastasis.

Metastasis of tumor is a multistep and complex process including local invasion, intravasation, survival in circulation, extravasation, micrometastasis formation and metastatic colonization [31]. For tumor cells metastatic programs, the expression of FOXM1 may regulate a series of interrelated events including the expession of caveolin-1 [23], VEGF [17,18], MPP-2 and MPP-9 [19], which are important to epithelial–mesenchymal transition (EMT), angiogenesis and metastasts. Importantly, a mounting body of work suggests that EMT, by which epithelial cells acquire mesenchymal characteristics leading to increased migratory and invasive potential of the cancer cells, has a critical role in metastasis [32,33]. Recently, numerous observations support the idea that the occurrence of EMT is closely associated with FOXM1 signaling activation. EMT has been reported as a pivotal program in several human solid cancers, including CRC [34], gastric cancer [25] and breast cancer [16], which is considered as the first step of tumor invasion and metastasis. Recent research shows that FOXM1c overexpression upregulates uPAR expression in pancreatic cancer cells and promotes EMT. Contrariwise, inhibition of FOXM1c expression suppressed uPAR expression by siRNA; furthermore, FOXM1c silencing expression could be targeted to reverse the acquiring of EMT phenotype in L3.7 cells [35]. Our present findings are consistent with these previous results. we transfected specific shRNA to FOXM1 into SW620 cells, which have a typical mesenchymal phenotype and high expression of FOXM1. We found that this transfection greatly inhibited FOXM1 mRNA and protein expression. Additionally, the SW620 cells reversed the acquisition of the EMT phenotype, changing from an elongated, spindle-shaped, mesenchymal phenotype to a more rounded, epithelial-like phenotype. As shown in recent studies, targeting FoxM1 signaling by novel small interference RNA silencing technique or miR-200 family members would be useful for reversing the EMT phenotype, which would likely result in the reversal of drug resistance and elimination of cancer cells [24]. Interestingly, knockdown expression of FOXM1 down-regulated mesenchymal cell markers Vimentin and Snail and up-regulated the epithelial cell marker E-cadherin, which acted as key regulators in the process of epithelial-to-mesenchymal transition (EMT). Emerging evidences suggest that many EMT-inducing transcription factors such as Snail and ZEB1 have been found to be associated with expression of FOXM1 [15,24,36], which leads to tumor aggressiveness and metastasis. E-cadherin is a typical epithelial marker and loss of E-cadherin is a significant hallmark of EMT and can be mediated by the repressor binding directly to E-box motifs within the proximal E-cadherin promoter, such as snail [37,38].

Snail is one of zinc-finger transcription factors that have been known as an essential player in the aggressive phenotype of EMT [39,40]. Increasing evidence suggests that Snail acts as a critical role during embryonic development and is essential for the formation of the fibroblasts during inflammation [41-43]. Moreover, Snail is highly expressed in CRC, especially in lymph node metastasis of CRC [44]. Consistent with our studies, FOXM1 has an intimate relationship with the expression of Snail in CRC cells. Recent studies demonstrated that ectopic FOXM1 activation increased Snail activity through AKT signaling in hepatocellular carcinoma cells isolated from FoxM1b Tg;Arf−/− mice. Increased FoxM1b levels induce EMT by activating AKT and subsequently increasing GSK-3β, which enhanced Snail expression [45]. In addition, new researches have verified that Foxm1 directly bound to and increased activity of Snail1 promoter, namely Snail1 is a direct downstream transcriptional target of Foxm1 in the radiation-induced pulmonary fibrosis and lung adenocarcinoma progression [43,46]. Taken together, our current study strongly suggests that FOXM1 signaling has important roles in CRC cells proliferation and aggressiveness; thus, it is indicated that an intervening strategy targeting FOXM1 signaling in colon cancer may be of clinical value.

## Conclusions

In summary, our studies have demonstrated that overexpression of FOXM1 is associated with distant lymph metastasis and tumor recurrence, and knockdown of FOXM1 by shRNA inhibited cell proliferation, migration and invasion of human colon cancer cells. In addition, we determined that downexpression of FOXM1 reversed the acquisition of EMT phenotype and upregulated the expression of E-cadherin, while decreasing Snail and Vimentin expression. Based on these studies, we conclude that FOXM1 may serve as a promising therapeutic target of CRC.
