# Correction to: Short hairpin RNA- mediated gene knockdown of FOXM1 inhibits the proliferation and metastasis of human colon cancer cells through reversal of epithelial-to-mesenchymal transformation

**DOI:** 10.1186/s13046-021-01918-6

**Published:** 2021-04-15

**Authors:** KanKan Yang, LinHua Jiang, You Hu, Jing Yu, HenFeng Chen, YiZhou Yao, XinGuo Zhu

**Affiliations:** 1 Department of General Surgery, The First Affiliated Hospital of Soochow University, Suzhou, 215006 Jiangsu Province China; 2 Department of Laparoscopic Surgery, The First Affiliated Hospital of Soochow University, Suzhou, 215006 Jiangsu Province China

**Correction to: J Exp Clin Cancer Res 34, 40 (2015)**

**https://doi.org/10.1186/s13046-015-0158-1**

Following publication of the original article [1], the authors identified minor errors in image-typesetting in Fig. 6; specifically in Fig. 6c.

The corrected figure is given below. The correction does not have any effect on the results or conclusions of the paper. The original article has been corrected.

**Fig. 6:**
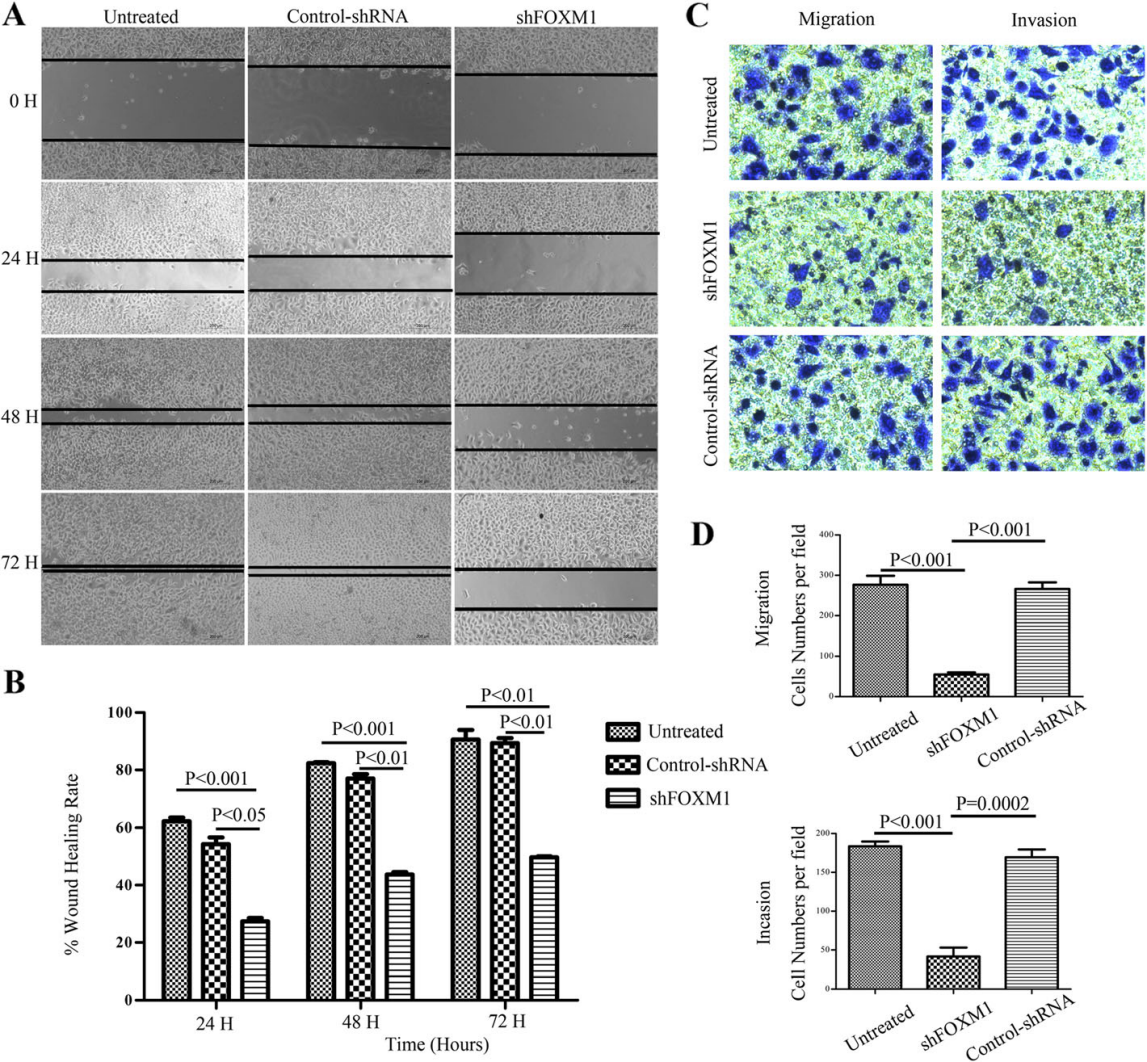
Effect of altered FOXM1 expression on colorectal cancer cell migration and invasion in vitro. **a**: Wound healing assays were carried out at 24 h after transfection in 24-well plates, when cell confluence rate reached above 90% and a linear wound across the monolayer was done. The wound gap was photographed every 24 h, the gap width was measured (μm) using Open Lab software. **b**: The wound rate was calculated and displayed graphically as described in the Materials and Methods. **c-d**: SW620 cells of three groups were digested and resuspended in serum-free culture medium and allowed to migrate toward the lower chamber with coated or uncoated matrigel for 24 h. Invading cells were stained with 0.1% crystal violet and counted manually. C-Left: transwell migration assay, Right: transwell invasion assay. **d**: The number of invading SW620 cells by cell migration (upper) and invasion (lower) assay was counted manually. Each experiment was repeated thrice independently. Scale bar = 200 μm in those figures
